# Clinical application of MOLLI T1* for extracellular volume calculation in healthy volunteers and aortic stenosis

**DOI:** 10.1186/1532-429X-17-S1-P11

**Published:** 2015-02-03

**Authors:** Anish N Bhuva, Thomas A Treibel, Arthur Nasis, Stefania Rosmini, Amna Abdel-Gadir, Heerajnarain Bulluck, Charlotte Manisty, James Moon

**Affiliations:** 1 The Heart Hospital Imaging Centre, London, UK

## Background

The calculation of the extracellular volume fraction (ECV) requires accurate quantification of myocardial and blood pool T1. Some Modified look locker inversion recovery (MOLLI) sequences provide a T1 and T1* output. T1* does not use a look locker correction, and so it is theoretically a more accurate estimation of true T1 blood T1 because fresh spins are flowing into the imaging plane. It is therefore recommended to use T1* for the quantification of the pre- and post-contrast blood pool. The aim of this study was to investigate the effect on ECV of using T1* (ECV_T1*_) rather than T1 (ECV_T1_) and assess accuracy, precision and bias.

## Methods

57 patients with aortic stenosis (AS) (mean age= 71±10 years, 33 female) and 25 healthy volunteers (HV) (mean age= 40±11 years, 19 female) were recruited. 4 chamber and mid ventricular short axis (SA) T1 maps were acquired pre-contrast and 15 minute post-contrast using 5s(3s)3s and 4s(1s)3s(1s)2s sequences respectively. Regions of interest (ROI) were drawn carefully to avoid blood-myocardium border and copied across series with correction only for patient movement. ECV was calculated as (Δ[1/T1_myo_] / Δ[1/T1_blood_]) * (1-haematocrit).

## Results

**Figure 1:**
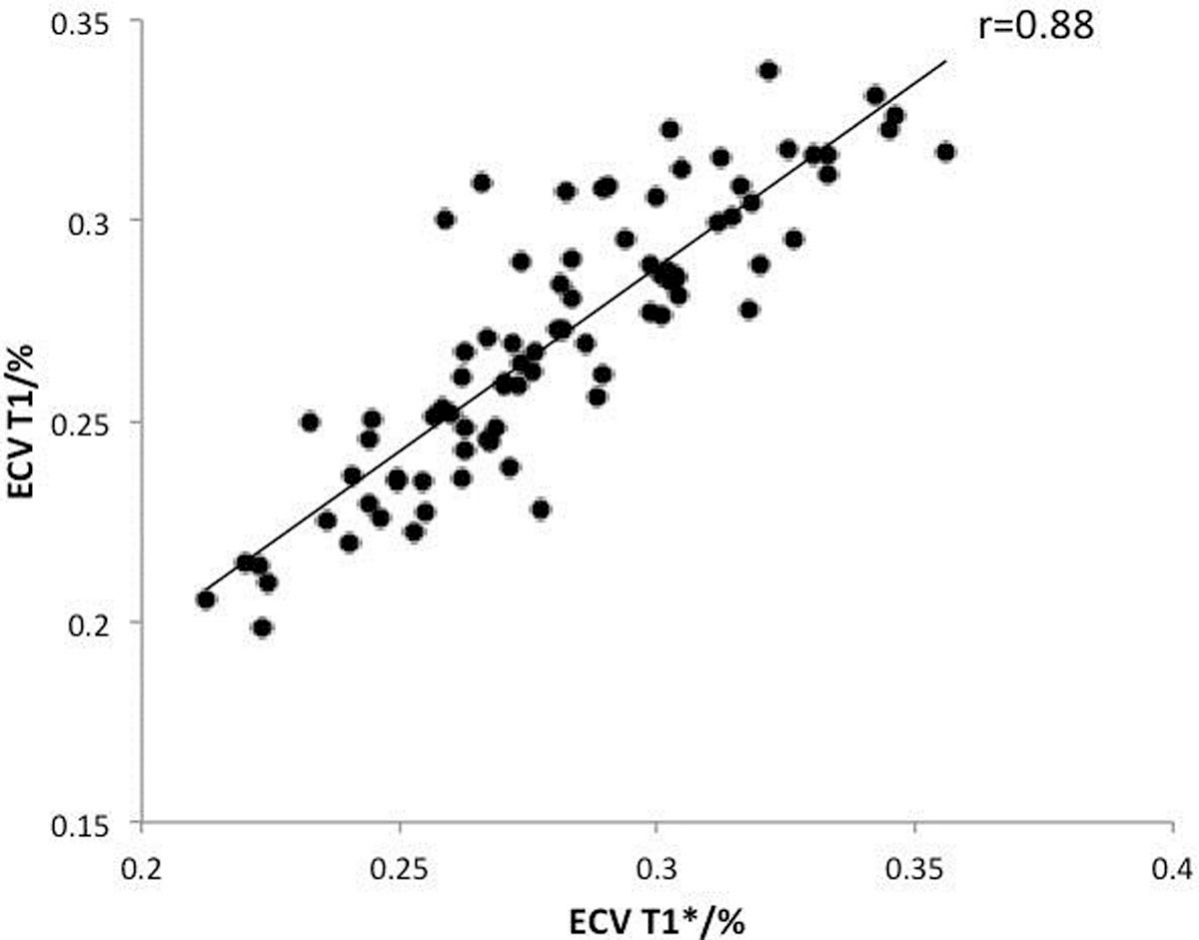
Excellent correlation between ECV BloodT1 and ECV BloodT1*

**Figure 2:**
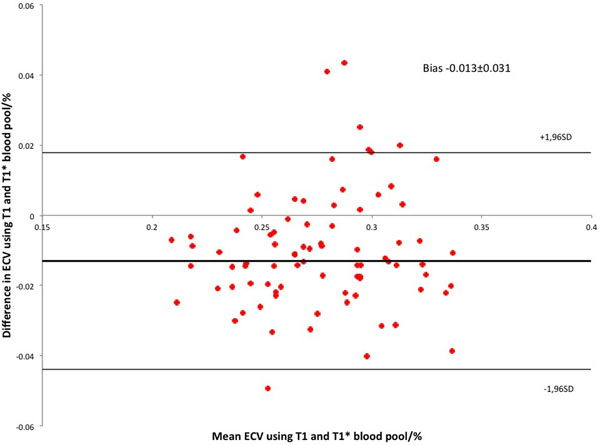
Little bias and variability between ECV BloodT1 and BloodT1* using Bland-Altman analysis

ECV_T1*_ was significantly lower than ECV_T1_ (mean 27.1±3.4% vs 28.1±3.2%, p<0.0001). ECV_T1*_ showed excellent correlation with ECV_T1_ (R= 0.88) (Figure 1). Bland-Altman analysis revealed no bias or variability (Figure 2). There was no statistical difference in variance between groups (F test, p= 0.66). In this group of subjects there was no difference in ECV between AS and HV groups using either ECV_T1_ (28.1±3.2% vs 28.2±3.4%) or ECV_T1*_ (27.3±3.6% vs 26.5±3.0%).

## Conclusions

ECV quantification using T1* can measure ECV across disease and normal populations, but its own normal values need to be referenced. It has similar variability, and no bias when compared to ECV using T1_blood_. ECV_T1*_ is therefore practically feasible and encourages further work to explore its theoretical accuracy by histological correlation.

## Funding

N/A.

